# Relative Value of Apothecaries' Weights in Different Countries

**Published:** 1836-04

**Authors:** 


### RELATIVE VALUE OF APOTHECARIES' WEIGHTS IN DIFFERENT COUNTRIES.

§ 1. In *England, Germany, Belgium, Sweden, Spain,* the medicinal *pound* is divided in 12 ounces; the *ounce* into 8 drachms; the *drachm* into 3 scruples; the *scruple* into 20 grains. The ounce, consequently, contains 480, and the pound 5760 grains.

§ 2. In *France,* the medicinal pound contains 16 ounces; the ounce 8 drachms; the drachm 72 grains.

In *Rome,* the medicinal pound contains 12 ounces; the ounce 24 *denari;* the denaro 24 grains.

In *Naples,* the medicinal pound contains 12 ounces; the ounce 10 drachms; the drachm 3 scruples; the scruple 20 *acini.*

§ 3. The following is the relative value of the medicinal pound in different countries, reduced to one standard, the French *gramme,* (equal to 0.06475 of the English grain.)

| | *Grammes.* |
|---|---|
| England, . . . . | 373.24* |
| France, . . . . | 489.50 |
| Austria, . . . . | 420 |
| Prussia, . . . . | 350.78 |
| Saxony, and most of the other German States, | 357.56 |
| Bavaria, . . . . | 360 |
| Belgium and Holland, . . | 375 |
| Sweden, . . . . | 356.29 |
| Poland, . . . . | 358.51 |
| Milan, . . . . | 420 |
| Naples, . . . . | 320.76 |
| Rome, . . . . | 339.13 |
| Spain, . . . . | 230 |

* 372.96, Edin. Dispens.

*Annalen der Pharmacie,* xii B. Heft 3.

